# Size and Composition of Caregiver Networks Who Manage Medications for Persons Living With Dementia: Cross-Sectional Analysis of the 2011-2022 National Health and Aging Trends Study

**DOI:** 10.2196/64499

**Published:** 2025-05-22

**Authors:** Reed WR Bratches, Frank Puga, Paul J Barr, Amanda N Leggett, Meredith Masel, James Nicholas Odom, Rita Jablonski

**Affiliations:** 1 School of Nursing, University of Alabama at Birmingham, 1701 University Blvd, Birmingham, AL, 35294, United States, 1 205 934 5428; 2 Center for Technology and Behavioral Health, Dartmouth College, Hanover, NH, United States; 3 Institute of Gerontology, Wayne State University, Detroit, MI, United States; 4 School of Public & Population Health, University of Texas Medical Branch, Galveston, TX, United States

**Keywords:** caregiver, dementia, disparities, cross-sectional analysis, racial, ethnic, family caregivers, logistic regression, secondary analysis, manage, management, medication, network

## Abstract

**Background:**

Family caregivers commonly help manage medications taken by persons living with dementia. Recent work has highlighted the importance of caregiver networks, which are multiple caregivers managing care for a single person, on managing care for persons living with dementia, especially medication management. However, less is known about the composition of caregiver networks.

**Objective:**

The objective of this analysis was to describe the composition of caregiver networks that manage medications, the factors associated with helping with medications within caregiver networks, and whether racial or ethnic differences exist in caregiver network composition.

**Methods:**

This cross-sectional secondary analysis used data from the National Health and Aging Trends Study (NHATS) “other person” files from 2011 to 2022. Descriptive statistics were calculated for caregivers who were identified as helping manage medications for a person with dementia. Mixed-effect logistic regression was used to determine factors associated with helping with medications among caregiver networks, with odds ratios converted to predicted probabilities using marginal standardization. A *P* value of .05 or less was considered statistically significant. Secondary analysis was stratified by race and ethnicity due to identified cultural differences in living situation and overall caregiver network composition.

**Results:**

A total of 15,809 caregivers were analyzed. Of those, 3048 (19.2%) managed medications for persons living with dementia. Caregiver networks that manage medications tend to include a spouse or partner and child, at least one of whom has a college degree. Every person with dementia reported at least 1 person who managed their medications. White persons with dementia had an average of 2.4 (range 1‐9) people who managed medications, while Black or African American persons with dementia had an average of 2.8 (range 1‐9) and Hispanic or Latino persons with dementia had an average of 2.9 (range 1‐8) people who managed medications. Spouses were most likely to manage medications across all racial and ethnic groups. In regression modeling, female gender (predicted probability [PP] 15%, 95% CI 13%-17%; *P*<.001), Black or African American race (PP 7%, 95% CI 4%-10%; *P*<.001), and Hispanic ethnicity (PP 4%, 95% CI 1%-9%; *P*=.04) were associated with an increased probability of helping with medications.

**Conclusions:**

The size and composition of caregiver networks that manage medications for persons living with dementia differ by race and ethnicity but typically includes at least 2 people, one of whom has a college degree. Helping with medications was more likely among non-White family caregivers, while White patients with dementia were more likely to use paid help to manage medications.

## Introduction

Persons living with dementia frequently take multiple medications due to comorbid conditions and pharmacologic attempts to manage the behavioral symptoms of dementia [1]. Family caregivers, or spouses, children, and friends who provide care for persons living with dementia, are often responsible for medication management [2]. Approximately 54% of family caregivers manage medications in early-stage dementia, rising to 90% in later dementia stages, as executive functioning declines and persons living with dementia become more reliant on family caregivers [3,4]. There are identified disparities in dementia diagnoses and medication management, dementia being more likely among Black and Hispanic persons [5], with family caregivers from historically minoritized racial and ethnic groups helping with medications at increased rates relative to non-Hispanic White caregivers [6,7].

Medication management of person living with dementia can be challenging and stressful for family caregivers [2,3,8]. Medication management includes components such as filling prescriptions and understanding medication purposes, organizing and administering medications, monitoring for side effects, and sustaining medication regimens, which can be very time-consuming for family caregivers [9].

Most prior studies have focused on medication management by family caregivers in the dementia dyad (eg, one person living with dementia and one family caregiver), but recent literature has highlighted the importance of caregiver networks in managing the care of persons living with dementia [10]. Caregiver networks can be defined as a cohesive network of family and friends who provide support to the persons living with dementia [11]. Caregiver networks can help reduce caregiver burden by sharing stressful tasks over multiple caregivers [12], and caregiver networks can facilitate better patient care through improved caregiver functioning [13]. Caregiver networks can be influenced by cultural factors including race and ethnicity [10]. For instance, Black and Hispanic persons living with dementia are more likely to rely on informal care from family and friends, live in a home with 3 or more household members, and have an adult child in the caregiving network than non-Hispanic White persons living with dementia [14,15]. Despite the importance of caregiver networks, current interventions to support medication management, like providing access to the medical record and using application-based medication trackers, are focused on the person living with dementia–caregiver dyad [8,16]. Networked interventions have the potential to improve adherence and reduce medication administration errors by including the full team of caregivers who help to manage medications [17].

While prior work has highlighted the importance of caregiver networks for providing care, and in communicating between members of caregiver networks when helping with medications [13], the characteristics of the caregiver networks that manage medications for persons living with dementia (the number of people helping with medications, the relationship between the persons living with dementia and the caregivers who help with medications, and the characteristics of those caregivers), and whether there are differences in the characteristics of those who manage medications for persons living with dementia by race and ethnicity, are currently unknown. Hence, the objective of this analysis was to describe the characteristics of caregiver networks that manage medications for persons living with dementia and determine what factors may be associated with helping with medications in caregiver networks.

## Methods

### Study Design and Setting

This cross-sectional secondary data analysis used data from the 2011 through 2022 National Health and Aging Trends Study (NHATS) “other person” files from rounds 1 through 12. The NHATS is an annual health-related study sampling Medicare beneficiaries in the United States [18]. The NHATS collects detailed information on participants’ physical and cognitive capacity, demographics, and living situations.

### Data Source

The NHATS “other person” file is constructed from a roster that is generated from questions in the main NHATS interview and survey, where NHATS respondents were asked about other persons who help with their care. Other persons are cumulative to NHATS participants across rounds, meaning additional other persons are added each round an NHATS participant is interviewed, and each other person is given a unique identifier and resampled with the NHATS participant. While the “other person” file does not specifically identify the demographic features of the NHATS participant, we derived this information by linking the “other person” files with the NHATS participant identifier. NHATS participants and caregivers were analyzed in their most recent year of participation, and data from all waves of NHATS were pooled to increase sample size and estimate the effect of the sampling round on medication management.

### Ethical Considerations

This analysis was determined as “not human subjects research” by the Institutional Review Board at the University of Alabama at Birmingham (#300013016). Analytic procedures were determined to be minimal risk to participants and did not require consent or contact with participants, and initial data collection for each NHATS round was approved by the conducting team. Data were deidentified prior to examination and analysis. The reporting of this study adheres to the RECORD (Reporting of Studies Conducted Using Observational Routinely Collected Data) statement (Checklist 1).

### Inclusion Criteria

For inclusion as a caregiver in our analysis, caregivers in the NHATS other person file must have been identified as a person who helps with medication management by the NHATS participant or their proxy. The caregiver in the NHATS other person file must also provide care to a patient with dementia, as defined in the NHATS Condition Variable 9, which asks about the presence or absence of a dementia diagnosis.

### Assessments

Demographic covariates for caregivers were selected from those collected in the NHATS other person file and included race, ethnicity, gender, relationship to persons living with dementia, education, age, and year of data collection. Race and ethnicity were categorized as White, Black or African American, Hispanic, or other, which included American Indian, Alaska Native, Asian, Native Hawaiian, and Pacific Islander.

Relationship to the persons living with dementia was categorized by self-report as sibling, child, grandchild, spouse or partner, paid helper, or other, which included the sons of step-siblings, boarders and renters, neighbors and nonrelated friends, nonrelated roommates, and ex-spouses or partners. A “paid helper,” according to the National Study of Caregiving (NSOC), is someone who was employed to provide care but was not an immediate family member.

We identified caregiver groups who help with medications as those in the other person file who help with the same patient’s medications, as determined by an affirmative response to the variable “MEDSHELP,” which asks whether the person helps with the patient’s medications.

### Study Size

We analyzed 15,809 unique caregivers from the “other person” files of NHATS rounds 1 through 12.

### Data Analysis

Mixed-effect logistic regression analysis was conducted based on the outcome variable “helping with medications” and independent demographic variables race, relationship to the patient, gender, and education, including a person living with dementia-level random effect to account for multiple caregivers caring for the same person living with dementia. As this was an exploratory rather than confirmatory analysis, variables were not added incrementally. To protect against overfitting and collinearity, we tested for collinearity between independent variables using the variance inflation factor (VIF) and defined problematic collinearity as a VIF greater than 10. We did not impute or otherwise include caregivers who provided missing data. We excluded covariates with problematic missingness, defined as having greater than 20% of missing observations, via listwise deletion to preserve sample size in the regression modeling [19]. Education and age were thus excluded from regression modeling due to missingness, though sensitivity analysis indicated no statistically significant difference between models including and excluding those variables. A predefined α level of .05 was used to determine statistical significance. Odds ratios were converted to predicted probabilities using marginal standardization to aid in interpretability [20]. All analyses were conducted in R version 3.4.1 (The R Foundation for Statistical Computing).

## Results

### Descriptive Results

Of the caregivers analyzed, 19.3% (3048/15,809) helped manage medications for persons living with dementia. Each person with dementia had at least 1 person who helped manage their medications. Caregivers who helped with medications were 56.9% (1633/2865) non-Hispanic White, 27.8% (799/2865) Black or African American, 9.8% (279/2865) Hispanic, and 4.7% (133/2865) other. Caregivers who helped with medications were 15.0% (445/3033) spouses, 39.1% (1184/3033) children, 4.9% (149/3033) grandchildren, 9.9% (303/3033) other relations, 30.4% (921/3033) paid help, and 1.5% (45/3033) siblings. The mean number of caregivers assisting a person living with dementia with their medications was 2.5, with a range of 1 to 9.

Among non-Hispanic White caregivers who manage medications (n=1633), the mean number of participants assisting with medications was 2.4 (range 1-9). Caregivers who helped with medications were 17.3% (282/1633) spouses, 33.9% (555/1633) children, 2.9% (48/1633) grandchildren, 9.6% (157/1633) others, 35.5% (580/1633) paid helpers, and 0.7% (11/1633) siblings.

Among Black or African American caregivers (n=799), the mean number of caregivers assisting with medications was 2.8 (range 1-9). Caregivers who helped with medications were 8.8% (71/799) spouses, 45.6% (365/799) children, 8.3% (67/799) grandchildren, 12.0% (96/799) others, 22.1% (177/799) paid helpers, and 2.6% (21/799) siblings.

Among Hispanic or Latino caregivers (n=279), the mean number of caregivers assisting with medications was 2.9 (range 1-8). Caregivers who helped with medications were 16.1% (45/279) spouses, 48.7% (136/279) children, 5.3% (15/279) grandchildren, 7.1% (20/279) others, 20.7% (58/279) paid helpers, and 1.7% (5/279) siblings.

Among “other” caregivers (n=133), the mean number of caregivers assisting with medications was 2.4 (range 1-7). Caregivers who helped with medications were 16.5% (22/133) spouses, 31.5% (42/133) children, 5.2% (7/133) grandchildren, 10.5% (14/133) others, 33.1% (44/133) paid help, and 3.0% (4/133) siblings.

Participant demographics can be found in Table 1. See Table 2 for more information on participants by race, ethnicity, and relationship.

**Table 1.** Participant characteristics.

	Did not help with medications (n=12,761), n (%)	Helped with medications (n=3048), n (%)	Total (N=15,809), n (%)
Race and ethnicity			
White	7166 (59.0)	1633 (57.4)	8799 (58.7)
Black	3242 (26.7)	799 (28.1)	4041 (27.0)
Hispanic	1183 (9.7)	279 (9.8)	1462 (9.8)
Other	552 (4.5)	133 (4.7)	685 (4.6)
Relationship			
Spouse or partner	284 (2.3)	445 (14.6)	729 (4.7)
Child	5452 (43.2)	1184 (38.9)	6636 (42.4)
Grandchild	1376 (10.9)	149 (4.9)	1525 (9.7)
Other	3789 (30.0)	303 (9.9)	4092 (26.1)
Paid help	809 (6.4)	921 (30.2)	1730 (11.0)
Sibling	906 (7.2)	45 (1.5)	951 (6.1)
Gender			
Male	2160 (48.9)	298 (25.0)	2458 (43.8)
Female	2259 (51.1)	895 (75.0)	3154 (56.2)
Education			
College degree	1180 (25.1)	339 (24.1)	1519 (24.9)
High school or some college	2941 (62.6)	908 (64.5)	3849 (63)
More than a college degree	580 (12.3)	161 (11.4)	741 (12.1)

**Table 2.** Number of caregivers helping with medications by race and ethnicity.

	White (n=1633)	Black (n=799)	Hispanic (n=279)	Other (n=133)
Caregivers, mean (range)	2.4 (1-9)	2.8 (1-9)	2.9 (1-8)	2.4 (1-7)
Relationship, n (%)				
Spouse or partner	282 (17.3)	71 (8.9)	45 (16.1)	22 (16.5)
Child	555 (34.0)	365 (45.7)	136 (48.7)	42 (31.6)
Grandchild	48 (2.9)	67 (8.4)	15 (5.4)	7 (5.3)
Other	157 (9.6)	96 (12.0)	20 (7.2)	14 (10.5)
Paid help	580 (35.5)	177 (22.2)	58 (20.8)	44 (33.1)
Sibling	11 (0.7)	21 (2.6)	5 (1.8)	4 (3.0)

### Statistical Results

In the univariate statistical model, gender (*P*<.001) and relationship (*P*<.001) were associated with helping with medications.

In the fully adjusted regression model, compared to non-Hispanic White race or ethnicity, Black race or ethnicity (predicted probability [PP] 7%, 95% CI 4%-10%; *P*<.001) and Hispanic race or ethnicity (PP 4%, 95% CI 1%-9%; *P*=.04) were associated with an increased likelihood of helping with medications among caregivers. Female gender (PP 15%, 95% CI 13%-17%; *P*<.001) was associated with an increased likelihood of helping with medications. Compared to a spouse or partner, children (PP −51%, 95% CI −56% to −46%; *P*<.001), grandchildren (PP −60%, 95% CI −66% to −54%; *P*<.001), paid help (PP −23%, 95% CI −39% to −7%; *P*=.006), and siblings (PP −63%, 95% CI −69% to −58%; *P*<.001) were all associated with decreased likelihood of helping with medications. See Table 3 for the fully adjusted regression modeling.

**Table 3.** Fully adjusted regression model predicting the probability of helping with medications.

	Predicted probability (95% CI)	*P* value
Female gender	0.15 (0.13 to 0.17)	<.001
Race and ethnicity		
White (reference)		
Black or African American	0.07 (0.04 to 0.1)	<.001
Hispanic	0.04 (0 to 0.09)	.04
Other	−0.01 (−0.07 to 0.05)	.8
Relationship		
Spouse or partner (reference)		
Child	−0.51 (−0.56 to −0.46)	<.001
Grandchild	−0.60 (−0.66 to −0.54)	<.001
Other	−0.62 (−0.67 to −0.57)	<.001
Paid help	−0.23 (−0.39 to −0.07)	.01
Sibling	−0.63 (−0.69 to −0.58)	<.001
Round	0 (0 to 0)	.96

### Sensitivity Analysis

We conducted a sensitivity analysis based on reducing the number of distinct relationship categories. In this model, which had relationship categories of spouse or partner, other relative, paid help, and other, we found no improvement from the full regression model when considering Akaike Information Criterion, Bayesian Information Criterion, or overall deviance. See Table 4 for sensitivity analysis.

In subgroup analysis stratified by race and ethnicity, female gender was associated with a higher likelihood of helping with medications, and compared to spouses and partners, children, grandchildren, paid help, and siblings were all associated with a lower likelihood of helping with medications. See Table 5 for subgroup analysis.

**Table 4.** Sensitivity results.

Model	AIC^a^	BIC^b^	Deviance
Full model	4415	4493	4391
Sensitive model	4457	4522	4437

a AIC: Akaike Information Criterion.

b BIC: Bayesian Information Criterion.

**Table 5.** Regression models stratified by race or ethnicity predicting the probability of helping with medications.

	Predicted probability (95% CI)	*P* value
White		
Female gender	0.12 (0.09 to 0.15)	<.001
Relationship		
Spouse or partner (reference)		
Child	−0.50 (−0.56 to −0.44)	<.001
Grandchild	−0.57 (−0.65 to −0.5)	<.001
Other	−0.61 (−0.68 to −0.55)	<.001
Paid help	−0.19 (−0.39 to 0)	.05
Sibling	−0.64 (−0.71 to −0.57)	<.001
Round	0 (−0.01 to 0)	.19
Black		
Female gender	0.21 (0.16 to 0.25)	<.001
Relationship		
Spouse or partner (reference)		
Child	−0.48 (−0.59 to −0.36)	<.001
Grandchild	−0.59 (−0.72 to −0.46)	<.001
Other	−0.59 (−0.72 to −0.47)	<.001
Paid help	−0.27 (−0.62 to 0.07)	.12
Sibling	−0.60 (−0.73 to −0.47)	<.001
Round	0 (−0.01 to 0.01)	.59
Hispanic		
Female gender	0.17 (0.11 to 0.24)	<0.001
Relationship		
Spouse or partner (reference)		
Child	−0.65 (−0.78 to −0.53)	<.001
Grandchild	−0.80 (−0.95 to −0.65)	<.001
Other	−0.76 (−0.91 to −0.6)	<.001
Paid help	−0.87 (−0.99 to −0.75)	<.001
Sibling	−0.61 (−0.89 to −0.33)	<.001
Round	0.01 (0 to 0.02)	.09
Other		
Female gender	0.17 (0.07 to 0.27)	<.001
Relationship		
Spouse or partner (reference)		
Child	−0.56 (−0.79 to −0.32)	<.001
Grandchild	−0.56 (−0.84 to −0.27)	<.001
Other	−0.58 (−0.84 to −0.33)	<.001
Paid help	0.30 (0.07 to 0.53)	.01
Sibling	−0.70 (−0.93 to −0.47)	<.001
Round	0.01 (−0.01 to 0.02)	.27

## Discussion

### Principal Results

In our analysis of 15,809 NHATS “other person” data records from 2011 to 2022, we found that the average person with dementia has 2.5 people that help with their medications, and that Black or African American and Hispanic persons with dementia have more people helping with their medications than White persons with dementia. Many children, grandchildren, and siblings of persons with dementia are helping with medications, in addition to spouses. In the regression modeling, we found that Black race, Hispanic ethnicity, and female gender were associated with an increased likelihood of helping with medications, while relationships other than spouses or partners were associated with a lower likelihood of helping with medications. We did not observe differences between helping with medications and the year of data collection.

### Limitations

This study was conducted using a large composite sample from a methodologically robust sample of Medicare beneficiaries. While we included participants from all sampled years (2011‐2022), we analyzed the sample cross-sectionally at the most recent year of data collection to ensure as large of a sample as possible, though our regression modeling is not generalizable to the broader population as we could not appropriately weigh our combined sample. We included sampling year as a covariate to minimize differences in year on our estimates and found that sampled year was not a statistically significant covariate for medication management, but future work should consider longitudinal trends in medication management by family caregiver networks. Additionally, we chose to exclude education level and age from our regression models due to missingness to preserve sample size on our primary covariates of interest. Prior work has not shown that education or age is associated with medication management among individual caregivers [7], and when our analysis included those variables, we found no statistically significant differences between the 2 models, but future work could consider whether those covariates affect helping with medications in caregiving networks. It is possible that the gender composition of our sample, which was 75% female and 25% male, may overestimate female gender being a statistically significant predictor in our regression modeling. Other work has highlighted the importance of including male dementia caregiver perspectives in research [21]; future targeted work to determine the effect of gender on predictors of network composition should consider oversampling male caregivers to limit this potential effect. Finally, we were unable to include the stage or severity of dementia, or what tasks are included in helping with medications, as this is not collected in NHATS. Future work should consider whether there are differences in caregiver networks that manage medications by stage of dementia, and identifying what tasks of helping with medications are being performed by different components of the network.

### Comparison With Prior Literature

Prior work with NHATS data from 2011 to 2015 has found that persons living with dementia have caregiver networks that assist with the tasks of care, which includes medication management as well as other medical-related tasks like attending clinic visits and interacting with providers [22]. They found that these networks range in size from 0 to 2+ caregivers but did not specify a total range. Our finding with data from 2011 to 2022 that caregiver networks included at least one person and, on average, 2.5 caregivers that manage medications indicates that medication management could be an activity that is more complex and requires more caregiver involvement than other caregiving tasks. The differences in network size that we found, while small, highlight the shared nature of caregiving. Not one group had an average of below 2.5 caregivers who manage medications, yet current methods of facilitating medication management are focused on an individual caregiver managing medications. Interventions to support medication management should consider supporting caregiver networks as well as the primary caregiver, as the average person living with dementia has at least 2 caregivers who help with medications. Potential interventions could include applications or integrations that allow for proxy access by caregivers or caregiver profiles that allow for the management of patient medications; these interventions have been considered by health systems, though not applied to medication management [23,24].

Other work on cultural differences in the composition of caregiver networks used data from NHATS from 2015 to 2019 and found differences in the size of caregiver networks by race and ethnicity. They found that Hispanic persons living with dementia had the largest care networks with 6.89 members, while non-Hispanic Black persons living with dementia had 6.69 members and non-Hispanic White persons living with dementia had 5.74 members [10]. Our results indicate that the size of the networks that manage medications are smaller than those that help with other tasks like transportation or cooking food, with Hispanic persons living with dementia having 2.9 members, non-Hispanic Black persons living with dementia having 2.8 members, and non-Hispanic White persons living with dementia having 2.4 members. The relative size of the caregiver networks that manage medications could be due to cultural factors: existing literature has shown differences in family size and cohabitation by race and ethnicity which can affect caregiver network size, and larger networks could have more caregivers helping with medications [14]. Network size could be influenced by living situation: Hispanic persons living with dementia and Black or African American persons living with dementia are less likely to live alone and more likely to live in multigenerational homes than non-Hispanic White persons living with dementia [25,26]. Multigenerational living situations could be affecting the size and composition of the networks of caregivers who help with medications.

Addressing cultural factors is critical in providing culturally specific interventions to support caregivers in medication management. Co-designed medication management materials, where interventions and protocol materials are developed collaboratively between persons living with dementia, caregivers, and researchers, have been identified as an informational gap by prior reviews [27]. Our study suggests that including multiple caregivers may be beneficial to co-designing future interventions, especially in Black and Hispanic communities that have more caregivers on average and different conceptualizations of the role of caregiving [28,29].

Our finding that spousal caregivers of persons living with dementia were more likely to manage medications when controlling for race and ethnicity and within each race and ethnicity extends prior work which found that spouses were more likely to manage medications for their spouses or partners in a sample that controlled for dementia status [30]. Spouses are more likely to live with the person receiving care and be near the person living with dementia around medication times [31]; however, it is notable that our analysis found that children and grandchildren are the plurality of caregivers who help with medications. While spousal caregivers could be most involved in the administration of medications in the home, children or grandchildren could be instrumental in filling or refilling medication prescriptions. Specific roles and aspects of helping with medications should be the target of additional future studies.

### Conclusions

Caregiver network size is an important consideration when designing interventions to support medication management by family caregivers, and this study sheds light on the size and composition of family caregiver networks that manage medications for persons living with dementia. Interventions to support medication administration for persons living with dementia should consider caregiver network size and composition to facilitate persons living with dementia adherence to medication prescriptions.

## Supplementary material

10.2196/64499 Checklist 1 The RECORD (Reporting of Studies Conducted Using Observational Routinely Collected Data) statement—checklist of items, extended from the STROBE (Strengthening the Reporting of Observational Studies in Epidemiology) statement.

## Abbreviations

NHATS: National Health and Aging Trends Study
PP: predicted probability
RECORD: Reporting of Studies Conducted Using Observational Routinely Collected Data
VIF: variance inflation factor
